# Folate, vitamin B12, ferritin and haemoglobin levels among women of childbearing age from a rural district in South India

**DOI:** 10.1186/s40795-017-0173-z

**Published:** 2017-06-26

**Authors:** Samiksha Singh, Jaga Jeevan Babu Geddam, G. Bhanuprakash Reddy, Dinesh Raj Pallepogula, Hira Ballabh Pant, Sutapa B. Neogi, Neena John, Sunanda Reddy Kolli, Pat Doyle, Sanjay Kinra, Andy Ness, Gudlavalleti Venkata Satyanarayana Murthy

**Affiliations:** 1 South Asia Centre for Disability Inclusive Development and Research, Indian Institute of Public Health-Hyderabad, Public Health Foundation of India, Plot No. 1, ANV Arcade, Amar Coop Society, Kavuri Hills, Madhapur, Hyderabad, Telangana 500033 India; 2 National Institute of Nutrition, Hyderabad, India; 3 Indian Institute of Public Health-Delhi, Public Health Foundation of India, Delhi, India; 4 South Asia Centre for Disability Inclusive Development and Research, Indian Institute of Public Health-Hyderabad, Centre for Applied Research and Education on Neurodevelopmental Impairments and Disability- related Health Issues (CARENIDHI), New Delhi, India; 5 London School of Hygiene and Tropical Medicine, London, UK; 6 NIHR Biomedical Research Unit in Nutrition, Diet and Lifestyle, University Hospitals Bristol Education Centre, Bristol, UK

**Keywords:** Folate, Folic acid, Vitamin B12, Ferritin, Haemoglobin, Deficiency, India, Women of childbearing age

## Abstract

**Background:**

Low folate and vitamin B12 levels have negative effect on pregnancy outcomes but there is paucity of data on their levels among Indian women. Ferritin and haemoglobin are associated with maternal mortality and low birth-weight. Our aim was to estimate the prevalence of deficiency of serum folate and vitamin B12, and low levels of serum ferritin and blood haemoglobin among women of childbearing age from a rural population of South India.

**Methods:**

We conducted a community-based cross-sectional study among 15-35 year women in a rural district. We used multistage stratified random sampling. Trained staff interviewed women to collect socio-demographic information and draw blood samples. We analysed samples for serum folate, vitamin B12, ferritin and blood haemoglobin levels and computed means and medians. We computed the proportion of deficiency based on cut-offs recommended by WHO. We examined the association of levels with age, parity and current pregnancy or breastfeeding by multi-variable regression using Stata 13.0.

**Results:**

We recruited 979 women. One-fifth (185, 19%) were pregnant and one-fifth (196, 20%)were breastfeeding. Median serum folate levels were 2.5 ng/ml (IQR, 1.2-4.8), median vitamin B12 levels were 228.0 pg/ml (IQR, 121 - 390), median ferritin levels were 13.0 μg/l (IQR, 6.0 - 20.0) and median blood haemoglobin levels were 12.1 mg/dl (IQR, 10.7 – 13.6). Low levels of serum folate, vitamin B12, ferritin and haemoglobin were found in 57% (95% CI, 54-60%), 44% (95% CI, 41-48%), 46% (95% CI, 43-49%) and 28% (95% CI, 25-31%) respectively. Women with folic acid deficiency had two times higher prevalence of having vitamin B12 deficiency. In adjusted regression analysis folate levels were lower in older and breastfeeding women, but not associated with parity and were higher among pregnant women. Similar associations were not found with Vitamin B12 deficiency. Ferritin levels were higher in older women; but not associated with parity, pregnancy or breastfeeding. Haemoglobin levels were lower in pregnant and breastfeeding women.

**Conclusion:**

Our findings suggest that folic acid, vitamin B12 and iron deficiency are important public health problems in India. We observed that half of the women of childbearing age were deficient in these nutrients. Folic acid and vitamin B12 deficiencies co-exist and should be supplemented together.

## Background

Vitamins such as folic acid and B12 play a key biological role in human reproduction [1] and development of the child [2]. Low folate and vitamin B12 levels in women are associated with megaloblastic anaemia [2] and, gestational hypertension and eclampsia [3] in the mother. Deficiency among mothers may increase the risk of many chronic and developmental disorders including neural tube disorders (NTDs) among their children [2, 4, 5]. Low folate level is an important determinant of spontaneous abortion, recurrent pregnancy loss, stillbirth [6–8], low birth weight [9–11] and preterm birth [10, 12, 13]. Low folate and vitamin B12 levels among a breastfeeding mother may result in poor cognitive functions of the child [2].

Folic acid and vitamin B12 deficiency are common globally, especially in low and middle-income countries (LMICs) [2, 14–17]. A recent systematic review observed that in a majority of countries where national surveys have been conducted, folic acid and vitamin B12 deficiency are public health problems [16]. Studies show that over the course of pregnancy, there is a steady decline in maternal plasma folate levels to about 50% of the non-pregnant levels [18, 19]. There is a paucity of data on folic acid and vitamin B12 deficiency among women in India. The few studies on pregnant women in India reveal that the prevalence of folic acid deficiency ranges between 20 and 30% [4, 20–22] and vitamin B12 deficiency between 50 and 80% [4, 23, 24], which is much higher than that reported from many other (LMICs) [25].

Iron deficiency is widespread across the world and is the most common cause of anaemia [26]. Iron deficiency and anaemia have effects on pregnancy outcome [27], and is known to be the most common contributing cause of maternal deaths in LMICs [28]. A recent systematic review found that daily prenatal use of iron and improvement in haemoglobin levels in pregnant women increased birth weight [29]. In India, four out of five pregnant women are found to be anaemic [30, 31] and many women experience adverse consequences [31].

Folate, vitamin B12 and iron deficiencies may co-exist and contribute to poor outcomes of pregnancy [25]. Serum levels of these nutrients shall be assessed through the stages of lifecycle (adolescent, pregnant and breastfeeding women) of young women to estimate patterns in the population and need of supplementation. The purpose of this study was to measure serum folate, vitamin B12, ferritin and haemoglobin levels, and to estimate the prevalence of deficiencies among 15-35 years women in a rural population of South-India.

## Methods

We conducted a population-based cross-sectional survey to assess the nutritional status of women between 15 and 35 years in the rural areas of Mahbubnagar district, Telangana India. Mahbubnagar has poor maternal and child health indicators and is one of the two high priority districts in the state [32].

The study was conducted over a period of 12 months from December 2012 to November 2013.

### Study population

All women in the age group 15-35 years, irrespective of their marital, pregnancy and breastfeeding status, residing in the study area at the time of the survey, were eligible for the study.

### Sample size

We used the estimates of folate and vitamin B12 for 20-40 year old adults in a study from South-India [33]. We calculated a sample size of 254 would be needed to estimate folic acid levels with a standard deviation of 3.1 ng/ml, absolute precision of 0.5 ng/ml and confidence interval of 99%. For vitamin B12, we calculated a sample size of 457 would be needed to estimate mean with a standard deviation of 166 pg/ml, absolute precision of 20 pg/ml and confidence interval of 99%. About 40% of the women in 15-35 years age in Telangana are likely to be pregnant or breastfeeding at any time. We increased the sample size to 1000 so that we have enough women who were i) neither pregnant nor breastfeeding and ii) those who were pregnant or breastfeeding at the time of the survey, in order to make comparisons.

### Sampling

We used a multi-stage stratified random sampling method. A district is divided into five revenue divisions for administrative purposes. We randomly selected one *mandal* (a smaller administrative unit) from each of the five revenue divisions. We listed all the villages in the sampled *mandals* and randomly selected 3 villages per mandal. Thus we selected 15 villages from 5 mandals of Mahbubnagar district. We listed households with 15-35 years married or unmarried women in each sampled village and randomly selected 30-35 households. We selected only one woman aged 15-35 per household. If there were more than one women per household, we selected one woman randomly. We included those who consented to the interview and collection of blood sample. The eligible women who were seriously ill (one woman had a high-grade fever and was bedridden) or refused to participate in the study (23 women) were excluded from the study.

### Data collection

Trained field officers elicited the socio-demographic and reproductive history of participants using a pre-tested semi-structured questionnaire and conducted anthropometric measurements. We developed a detailed operations manual for blood collection, handling, and transportation. A trained lab technician collected about 10 ml whole blood in EDTA vials/vacutainers under aseptic conditions and centrifuged the sample in the field within 6 h and stored in the refrigerator in the local lab for almost a day. The samples were transported to NIN labs in Hyderabad (100 km) three times per week for testing.

### Measurements

Blood tests: Levels of plasma folate and vitamin B12 were measured using the solid phase radioimmunoassay method as reported earlier [33] with a commercially available kit designed for simultaneous measurement of folic acid and vitamin B12 (Siemens Medical Solutions Diagnostics, Los Angeles, USA). Serum ferritin was estimated using an immune radiometric assay system with an available (^125^I) kit provided by Institute of Isotope Ltd. Blood haemoglobin was estimated using Cyanomethaemoglobin estimation method [34].

We also estimated serum albumin, total protein and vitamin A levels along with anthropometric measures to have an understanding of women’s general nutritional status. We measured weight (kg) and height (cm) (in order to calculate Body mass index (BMI) kg/mt.sq) and skin fold thickness (cms) from four sites- biceps, triceps, subscapular and supra-iliac areas.

### Definitions

Folate deficiency was defined as <3 ng/ml, vitamin B12 deficiency as <203 pg/ml, low ferritin as <12 ng/ml, anaemia as haemoglobin <11 g/dL, low protein as <6.4 g/dl, low albumin as <3.5 g/dl, and low vitamin A as >20 μg/dl [2, 35]. These were based on cut-offs recommended by WHO.

### Ethics approval

The ethics committee of Public Health Foundation of India (TRC-IEC-117/11) and the National Institute of Nutrition granted ethical approval for this study. A written informed consent was obtained from all the women included in the study. In the case of minors, consent was obtained from one of the parents or guardians.

### Statistical analysis

The analysis was conducted using STATA 14.0 SE. We computed mean and median levels of nutrients and the proportion of people with deficiency or low levels of nutrients under study. We estimated median and proportions for age group, parity and pregnancy/lactation status to study patterns. We developed histogram for each of the nutrients and found that serum folate, vitamin B12 and ferritin levels had skewed distribution. So we also estimated geometric means for these nutrients. We used log-transformed values to test for association with factors such as age, parity and current pregnancy or breastfeeding status using multivariable linear regression (model-1). We also conducted regression analysis (model-2) adjusting additionally for serum protein and BMI levels. We did not find any significant association of protein and BMI levels with the four main nutrients under study. The pattern and magnitudes of effect in model-1 did not change in model-2, so we did not report model-2.

## Results

A total of 979 women in age 15-35 years were included, of whom 19% were pregnant and 20% were breastfeeding at the time of the survey. (Table 1)

**Table 1:** Socio-demographic characteristics of participants

		Total	Percent
		979	100
Age group	15-19	308	31.5
	20-24	338	34.5
	25-29	199	20.3
	30-35	134	13.7
Married	Unmarried	308	31.5
	Married	656	67.0
	Widowed/divorced	15	1.5
Currently pregnant/lactating	Currently pregnant	185	18.9
	Currently Lactating	196	20.0
	Others	598	61.1
Parity	0	438	44.7
	1	203	20.7
	2to3	303	30.9
	>3	29	3.0
	Not available	5	0.6
History of still-birth		28	2.9
History of abortion		99	10.1
Age at marriage	<18 years	381	38.9
	18 or more years	286	29.2
	Don’t know	4	0.4
	Unmarried	308	31.5
Age at first childbirth	<19 years	212	21.7
	19 or more years	328	33.5
	Don’t know	1	0.1
	No birth	438	44.7

**Fig. 1:**
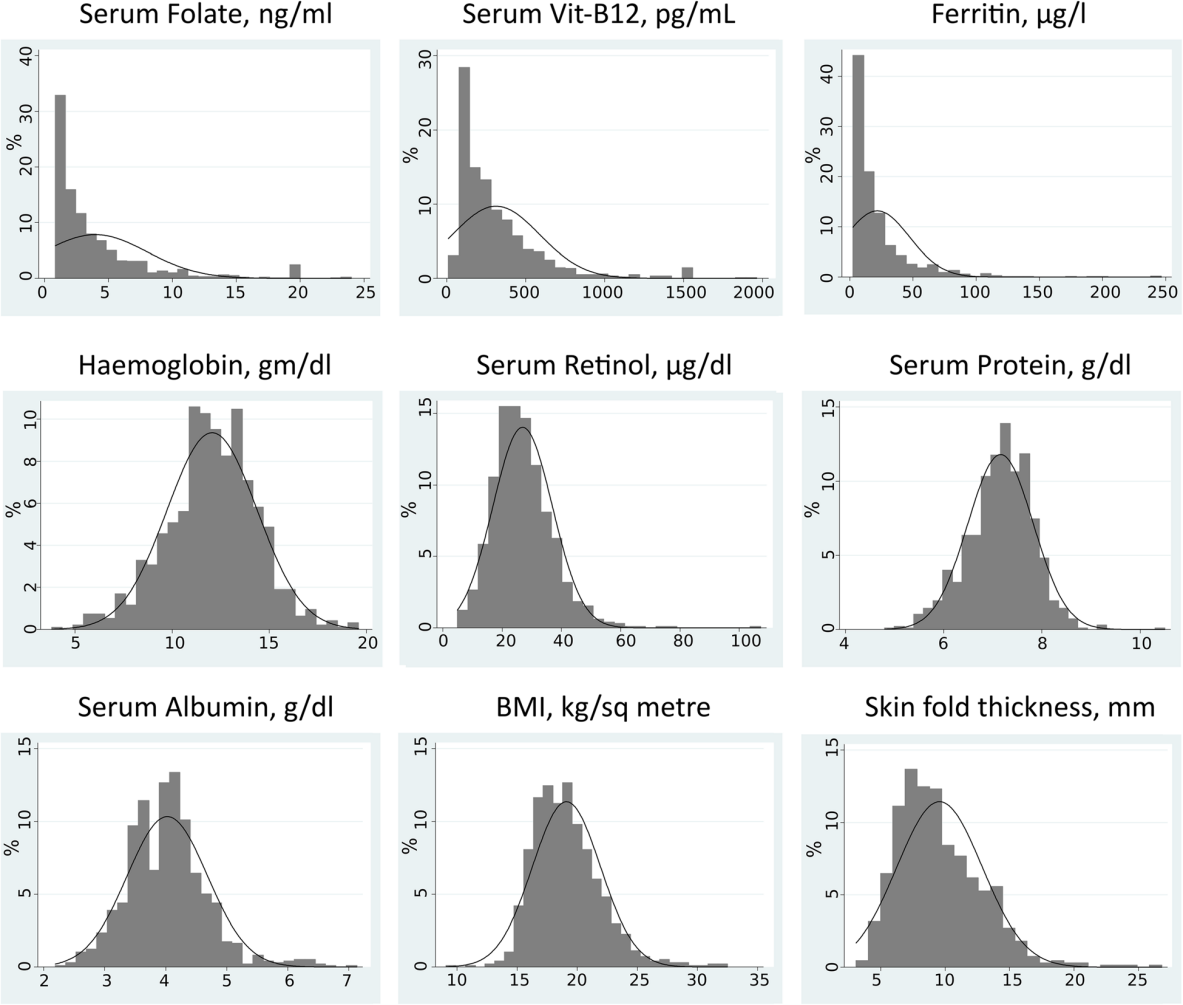
Distribution curves for studied nutrition parameters

The distribution curves for the nutrients under study are shown in Fig. 1 and, their mean and median serum/blood levels are shown in Table 2. Distribution curves for serum folate, serum vitamin B12 and serum ferritin levels were skewed and closer to zero. Distribution curves for blood haemoglobin, serum vitamin A, serum protein, serum albumin, BMI and total skinfold thickness (four sites) were near to normal distribution.

**Table 2:** Means and Median values of selected nutrition parameters

	Normal^a^	Mean (95% C.I.)	Median (IQR)	Deficiency% (95% C.I.)
Serum Folate in ng/ml	≥3	4.0 (3.7-4.2)	2.5 (1.2-4.8)	56.8 (53.7 – 59.9)
Serum Vit-B12 in pg/mL	≥203	314.4 (296.6-332.1)	228 (121.0-390.0)	44.4 (41.3 – 47.6)
Serum Ferritin in ng/ml	≥12	21.4 (19.8-23.0)	13.0 (6.0-20.0)	46.3 (43.2 – 49.4)
Blood Haemoglobin in gm/dl	≥11	12.1 (11.9-12.2)	12.1 (10.7-13.6)	28.4 (25.6 – 31.4)
Serum Retinol (Vit-A) in μg/dl	≥20	26.9 (26.3-27.5)	25.7 (20.2-32.5)	23.9 (21.3 – 26.7)
Serum Protein g/dl	6.4-8.3	7.17 (7.12-7.21)	7.2 (6.8-7.6)	12.2 (10.3 – 14.4)
Serum Albumin g/dl	3.5-5.0	4.01 (3.97-4.05)	4.0 (3.6-4.4)	18.6 (16.3 – 21.2)
BMI in kg/sq. metre	18-25	18.6 (18.4-18.8)	18.8 (17.1-20.7)	45.6 (42.5 – 48.7)
Mean Skin fold thickness in mm	-	9.6 (9.3-9.8)	9.0 (7.1-11.5)	-

^a^reference from WHO

Women aged 15-35 years had median serum folate levels of 2.5 ng/ml (IQR, 1.2 - 4.8), median serum vitamin B12 of 228 pg/ml (IQR, 121 - 390), median serum ferritin of 13.0 μg/l (IQR, 6.0 - 20.0) and median haemoglobin of 12.1 g/dl (IQR, 10.7 – 13.6). Mean and median values for nutrients are shown in Table 2. Only median level of folic acid was lower than the recommended cut-off. All other values for all the nutrients under study were above WHO cut-offs.

A large percentage of women were found to have nutritional deficiencies or low levels of ferritin or haemoglobin (Table 2). Folate deficiency was found in 56.8% (95% C.I. 53.7-59.9) women, vitamin B12 deficiency in 44.4% (95% C.I. 41.3-47.6), low serum ferritin in 46.2% (95% C.I. 43.2-49.4) and low haemoglobin levels in 28.4% (95% C.I. 25.3-31.4) of women. About one-half women were found to be underweight. Women with folate deficiency had roughly two times higher prevalence of vitamin B12 deficiency, while no substantial association was observed with ferritin deficiency or low Haemoglobin levels.

Median serum folate levels and prevalence of nutrients with respect to age group, parity and current pregnant/breastfeeding status are described in Table 3. Geometric means and regression analysis results are showed in Table 4.

**Table 3:** Serum folate, serum vitamin B12, serum ferritin and blood haemoglobin levels and deficiency with age, parity and current pregnancy and lactation status

	Folate, ng/ml	Vitamin B12, pg/ml	Ferritin, ng/ml	Haemoglobin, gm/dl
Median (IQR)				
Age group				
15-19	4.1 (2.7 – 6.4)	225.0 (140.0 – 367.5)	6.0 (5.0 – 15.0)	12.5 (10.8 – 13.8)
20-24	1.9 (1.0 – 4.3)	232.5 (119.8 – 232.5)	16.4 (9.1 – 31.6)	12.0 (10.7 – 13.5)
25-29	1.8 (1.0 – 4.4)	228.0 (107.0 – 435.0)	14.7 (8.1 – 26.4)	12.1 (10.7 – 13.6)
30-35	1.8 (1.0 – 2.8)	228.5 (100.0 – 400.3)	17.3 (8.7 – 31.6)	11.9 (10.3 – 13.5)
Parity				
0	3.7 (2.3 – 6.0)	220.0 (126.0 – 372.0)	8.3 (5.0 – 20.5)	12.1 (10.6 – 13.6)
1	2.1 (1.2 – 4.8)	235.0 (121.0 – 418.0)	17.6 (9.4 – 28.0)	11.8 (10.6 – 13.5)
2to3	1.6 (1.0 – 3.0)	235.0 (120.0 – 421.0)	16.0 (8.6 – 32.9)	12.4 (11.1 – 13.7)
> 3	1.0 (1.0 – 2.5)	257.0 (101.0 – 468.0)	15.4 (9.1 – 20.1)	11.8 (10.1 – 13.7)
Current status				
Not pregnant/lactating	2.8 (1.5 – 4.7)	226.0 (120.0 – 390.0)	9.4 (5.0 – 22.0)	12.2 (10.7 – 13.7)
Currently pregnant	4.3 (2.1 – 10.0)	218.0 (119.0 – 395.5)	15.0 (8.8 – 30.1)	11.3 (9.9 – 12.6)
Currently lactating	1.3 (1.0 – 2.1)	250.5 (131.3 – 385.5)	19.3 (11.2 – 37.9)	12.7 (11.4 – 13.9)
Deficiency % (95% C.I.)				
Age group				
15-19	31.8 (26.8 – 37.2)	45.1 (39.6 – 50.7)	68.5 (63.1 – 73.5)	26.9 (22.3 – 32.2)
20-24	66.6 (61.4 – 71.4)	43.8 (38.6 – 49.1)	34.0 (29.2 – 39.3)	29.2 (24.4 – 34.4)
25-29	64.8 (57.9 – 71.2)	45.2 (38.4 – 52.2)	40.9 (34.3 – 47.9)	28.8 (22.8 – 35.6)
30-35	77.6 (69.7 – 83.9)	43.3 (35.1 – 51.8)	33.8 (26.3 – 42.3)	29.7 (22.4 – 38.2)
Parity				
0	40.2 (35.7 – 44.9)	46.8 (42.2 – 51.5)	61.1 (56.3 – 65.4)	29.4 (25.2 – 33.9)
1	61.6 (54.7 – 68.0)	43.3 (36.7 – 50.3)	30.5 (24.6 – 37.2)	31.4 (25.2 – 38.4)
2	74.9 (69.7 – 79.5)	42.6 (37.1 – 48.2)	36.5 (31.3 – 42.2)	24.1 (19.5 – 29.3)
> 3	82.8 (64.3 – 92.8)	37.9 (22.1 – 56.8)	44.8 (27.8 – 63.1)	37.9 (22.2 – 56.8)
Current status				
Not pregnant/lactating	54.0 (50.0 – 58.0)	45.0 (41.0 – 49.0)	55.7 (51.7 – 59.7)	27.8 (24.3 – 31.5)
Currently pregnant	36.8 (30.1 – 44.0)	45.9 (38.9 – 53.2)	36.8 (30.1 – 44.0)	42.1 (34.9 – 49.7)
Currently lactating	84.2 (78.4 – 88.7)	41.3 (34.6 – 48.4)	26.5 (20.8 – 33.2)	18.1 (13.2 – 24.3)

**Table 4:** Geometric mean of serum folate, serum vitamin-B12, serum ferritin and blood haemoglobin levels and regression analysis adjusted for age, parity and current pregnancy and lactation status

	Folate		Vitamin-B12		Ferritin		Haemoglobin	
	Geometric Mean (95% C.I.)	Adjusted coefficient^a^ (95% C.I.)	Geometric Mean (95% C.I.)	Adjusted coefficient^a^ (95% C.I.)	Geometric Mean (95% C.I.)	Adjusted coefficient^a^ (95% C.I.)	Arithmetic Mean (95% C.I.)	Adjusted coefficient^b^ (95% C.I.)
Age group								
15-19	4.1 (3.8 - 4.3)	0	214 (196 - 233)	0	9.3 (8.5 - 10.1)	0	12.1 (11.9-12.4)	0
20-24	2.4 (2.2- 2.6)	−0.74* (−0.90 - -0.58)	241 (222 - 260)	0.04 (−0.14 – 0.22)	18.0 (16.4 - 19.6)	0.44* (0.25 - 0.63)	12.0 (11.8 – 12.3)	−0.35 (−0.89 - 0.19)
25-29	2.4 (2.1 - 2.7)	−0.63*(−0.81 - -0.44)	247 (222 - 276)	0.04 (−0.17 – 0.24)	15.7 (14.0 - 17.7)	0.38* (0.15 – 0.60)	12.0 (11.6 – 12.4)	−0.54 (−1.17 - 0.08)
30-35	1.9 (1.7 - 2.1)	−0.73* (−0.93 - -0.53)	234 (207 - 266)	−0.02 (−0.24 – 0.20)	17.2 (14.8 - 20.1)	0.53* (0.29 – 0.78)	12.0 (11.6 – 12.4)	−0.59 (−1.27 - 0.09)
Parity								
0	3.7 (3.4 - 3.9)	0	216 (200 - 232)	0	10.9 (10.1 – 11.9)	0	11.9 (11.7-12.2)	0
1	2.7 (2.3 - 3.0)	0.04(−0.12 – 0.20)	248 (223 - 276)	0.12 (−0.06 – 0.29)	17.6 (15.8 - 19.6)	−0.01 (−0.20 – 0.18)	11.9 (11.6-12.3)	0.14 (−0.41 - 0.69)
2 or more	1.9 (1.8 - 2.1)	−0.0(−0.24 – 0.09)	246 (227 - 267)	0.13 (−0.06 – 0.31)	17.0 (15.5 - 18.7)	−0.02 (−0.17 – 0.22)	12.3 (12.0-12.6)	0.49 (−0.07 - 1.05)
Current status								
Not pregnant/breastfeeding	2.8 (2.6 - 3.0)	0	225 (212 - 240)	0	11.7 (10.9 – 12.5)	0	12.1 (11.9-12.3)	0
Pregnant	4.5 (3.9 - 5.1)	0.81*(0.67 – 0.95)	239 (214 - 267)	0.01 (−.15 – 0.16)	17.7 (15.7 – 19.9)	0.23 (0.06 – 0.40)	11.3 (11.0-11.6)	−0.57** (−1.05 - -0.09)
Breastfeeding	1.6 (1.5 - 1.8)	−0.17**(−0.32 - -0.03)	248 (224 - 274)	−0.01 (−0.17 – 0.15)	20.4 (18.2 – 22.8)	0.36* (0.18 – 0.53)	12.8 (12.5-13.0)	0.68** (0.19 - 1.16)

^a^Regress Ln X age_group parity current_status; ^b^ Regress X age_group parity current_status; * *p* value <0.001

The proportion of folate deficiency was higher as age increased while ferritin deficiency was lower. Folate deficiency was higher as multiparity increased while ferritin deficiency was highest in nulliparous and high in grand multiparous women. The proportion of folate deficiency was lowest, ferritin was low but the proportion of anaemia was highest among currently pregnant women. Proportion of anaemia did not vary substantially with age or multiparity. Proportion of vitamin B12 deficiency did not vary with any group.

After adjustment for age group, parity and pregnancy/breastfeeding status, we observed that folate levels were low in older women (*p* < 0.001), but there was no association with parity. Pregnant women had higher folate levels and breastfeeding women had lower folate levels compared to non-pregnant/breastfeeding women (*p* < 0.001).

After adjusted analysis, serum vitamin B12 levels were not associated with age group, parity or pregnancy/breastfeeding status. Serum ferritin levels were higher for older, pregnant and breastfeeding women but were not associated with parity. Blood haemoglobin levels were lower among pregnant and breastfeeding women (*p* = 0.019 and 0.006).

## Discussion

In our study, nearly half women had folate, vitamin B12 and iron deficiency. These proportions were higher compared to other studies from India [4, 20–22, 33, 36] and other low-middle income countries [25]. Furthermore, in our study women with folic acid deficiency were two times more likely to have vitamin B12 deficiency compared to women without folic acid deficiency. Our study population was mostly rural from a poor district. It is likely that women in our study had poor diet contributing to high proportion of folic acid and vitamin B12 deficiency.

We observed that after adjusting for parity and age, folate and ferritin levels were higher in pregnant women compared to non-pregnant women. Most pregnant women receive iron-folic acid supplementation during pregnancy thus they may have higher serum levels of these, opposite to what is physiologically expected. Vitamin B12 deficiency is comparatively higher in adolescents and continues to be so in other stages. Studies among adolescents [37] and non-pregnant women [38] from other parts of India report a high prevalence of vitamin B12 deficiency but no folic acid deficiency. Studies of pregnant women in India have reported lower proportions of folic acid deficiency (20-30%) but higher proportions of vitamin B12 deficiency (50-80%) compared to our study [4, 23, 24]. Evidence from several studies suggests that folic acid and vitamin B12 deficiency are widespread and common in India.

Despite the high prevalence of folic acid, vitamin B12 and iron deficiency in this population, anaemia was observed only in 28% women which was lesser than estimates for women from the state of Telangana (57%) [39]. A review found that biochemical deficiency of folic acid and vitamin B12 does not translate into comparable prevalence of anaemia due to iron deficiency, malaria etc. [40] Our study district had a low prevalence of malaria compared to other tribal areas in the state thus may have a low prevalence of anaemia. Role of high prevalence of folic acid and vitamin B12 deficiency during pre- and peri-conception period shall be assessed by studying burden of adverse outcomes of pregnancy such as NTDs [2].

In our study, we found that low folate levels were associated with older age and pregnancy and breastfeeding status but not with parity. A survey in urban adult population in South India also observed an age-related decrease in folic acid levels [33]. The folic acid deficiencies are likely to worsen with subsequent pregnancies [41] especially where the birth interval is short [42]. Vitamin B12 levels were not found to be associated with age, parity or current pregnancy and breastfeeding status.

A study among adults in South-India found that 66% and 40% of the study population was meeting 70% of the RDA for folic acid and vitamin B12 [33]. This study found a correlation between the plasma status and the dietary intake of vitamin B12, while the plasma status and the dietary intake of folic acid were not correlated [33]. In India, women with confirmed pregnancy are given 5 mg folic acid tablets daily till 3 months of pregnancy after which they are provided iron-folic acid tablets. But due to unawareness about folic acid supplementation and since most of the pregnancies are unplanned, women contact health care providers in the first trimester or later.

Folic acid and vitamin B12 deficiency during pregnancy lead to maternal hyperhomocysteinemia which is associated with adverse outcomes of pregnancy. In the predominantly vegetarian Indians who eat only small amounts of animal-derived foods [33, 43], hyperhomocysteinemia is usually associated with vitamin B12 deficiency [44–46]. A study from India found that vitamin B12 supplementation but not folic acid was associated with lower plasma total homocysteine concentration [4].

Given the evidence on folic acid and vitamin B12 deficiencies from India (including the current study), a public health intervention shall address nutritional deficiencies for all stages of the women’s life-cycle along with associated social and developmental factors [25, 47]. All women planning pregnancy and, pregnant and breastfeeding mothers in India, especially from tribal and rural populations, should have access to nutrients rich food and supplementation [2, 5, 48]. Several reviews and guidelines have prescribed dosage for folic acid supplementation but there are no dosages identified for vitamin B12 supplementation [5]. Even at the prescribed dosages, monitoring of the consequences of folic acid and vitamin B12 intake are required to measure harmful effects of these at high concentrations [5, 49].

Our study provides community-based evidence for folic acid and vitamin B12 deficiency among non-pregnant, pregnant and breastfeeding women, and assessed the pattern across these stages adjusting for age and parity. The levels in non-pregnant women shall reflect preconception folate and vitamin B12 levels and thus help while planning and follow-up of nutrition interventions. We did not do a dietary assessment and thus were not able to associate findings with nutritional uptake. We did not capture information on gestational age thus couldn’t assess association with trimesters in pregnancy. Our study was in a predominantly rural and poor population thus results are not generalizable to the whole of India. Further research is required to assess the association of these deficiencies with adverse pregnancy outcomes in India and define the cut-offs for Indian population.

## Conclusions

One in two women in the childbearing age group is deficient in folate, vitamin B12 and iron in rural South-India. Anaemia was found in one in four women. Never pregnant women had a high proportion of vitamin B12 deficiency but not folic acid. Folate deficiency increased with age. Our findings confirm that folate and vitamin B12 deficiency is a public health problem in rural women of childbearing age. As these deficiencies are likely to lead to poor foetal and maternal outcomes, nutrition supplementation programs should focus on providing these micronutrients, especially to women in a child-bearing age in high folic acid and vitamin B12 deficient populations. As folic acid and vitamin B12 deficiencies co-exist they should be supplemented together. However, doses of folic acid and particularly vitamin B12 and their side effects at higher doses will need to be monitored.

## Abbreviations

BMI: Body mass index
IQR: Inter-quartile range
LMIC: Low and middle-income country;
NTD: Neural tube disorder
